# Graduate Study Abroad

**Published:** 1909-09-04

**Authors:** 


### Post-Graduate Medical Study.

### GRADUATE STUDY ABROAD.

On more than one occasion we have laid stress in the columns of The Hospital, on the importance, for medical practitioners, of becoming acquainted with the diverse views and opinions held by foreign colleagues, and of knowing something of the various methods of treatment and diagnosis adopted abroad. Such acquaintance and such knowledge may be gained by the study of foreign professional journals, monographs, and papers; and nowadays, when what is best and most useful is generally to be found translated into English, it is not difficult, even for those who are unacquainted with any language but their own to master, at least superficially, the principles of new methods of treatment emanating from Continental schools. To instance one example, the Bier method of treatment is now sufficiently well known in England to make it possible for a practitioner to obtain a working knowledge of its application without being obliged to leave England and study its working at Bonn or Berlin. Again, the specific reactions with tuberculin attenuations which go by the name of Calmette and Von Pirquet are equally well known: they are being tried and studied in England at nearly every hospital. But for those who wish to gain not merely a cursory insight into foreign methods but a real knowledge of what is best and most worthy of imitation in the systems in vogue in Continental schools a visit to these schools is of far greater real educative value than is the mere reading of reports about what is being done there. Once more to instance an example, however much may be written about the Bier method of treatment the graduate will learn its essentials far more quickly and far better by a few attendances at the Konigliche Chirurgische Klinik in the Ziegelstrasse Berlin, where Professor Klapp, Professor Bier's first assistant, personally supervises the treatment, than by reading all that has been written on the subject by enthusiastic supporters and as vehement opponents.

### The Advantages of Graduate Study Abroad.

To the graduate who desires to improve his knowledge and enlarge his purview, there is nothing better than a course of study abroad. No stress need here be laid upon the pleasures concomitant to such peregrinations in pursuit of knowledge. Everyone who has spent a year or more of his time in visiting continental clinics knows how many friends he has gained during his wanderings, for the Studienreise always brings the student into touch with men whom he learns to respect, to admire, and to love. It is an interesting discussion this: how much more conducive to real friendship is this intercourse between graduate student and graduate teacher than between undergraduate and undergraduate lecturer. Only in rare cases is the latter the means of framing a lifelong friendship and real attachment between the teacher and the taught. Otherwise is the case with the graduate student, for reasons that are obvious. He approaches his teacher on a more or less equal footing as a man; the raised level is made by superior skill and experience, both of which are his to acquire. Where tastes are mutually in agreement, he finds a common platform for extra professional discussion, and in matters of purely professional interest he may realise that the acquaintance he has made is on a more solid, a more equitable basis than when he became familiar with his old hospital teacher.

### A Liberalising Factor.

But it is not the personal element, however large and valuable a factor that may be, that counts in estimating the importance of graduate study abroad. There is further the general advantages it offers the student in broadening his mind liberalising his ideas, expanding his thoughts, strengthening his opinions. He becomes a " studied man " in the true sense of the term. Obviously the man who has a practical acquaintance with three methods is better able to judge the merits of one than he who is unfamiliar with any except his own pet method which as a student he was taught in the schools. The one school medical man is apt to be biassed, to be truly insular in his prejudices and his likes. The travelled graduate on the other hand, if he has made good use of his opportunities, is able to judge with discrimination and to compare with tact. His experiences among his foreign colleagues have taught him that there are more Meccas than one, and that his Koran does not hold the only true script of salvation.

### Recent and Future Developments.

In preceding issues of The Hospital we have given in detail such information as may be of use in helping the graduate student who is desirous of going abroad for purposes of study, to make his choice, of hospital, clinic, or town. It is, therefore, unnecessary to give these details here; for those who wish further information on the subject we refer to past issues of this paper. It is our intention to devote special columns to the graduate student and his work, and to give from time to time articles of a helpful and informative character. Meanwhile it is worth noting that great things are promising in the field. The present International Congress of Medicine now sitting at Budapest will discuss the question of the internationalising of graduate study, and it is hoped that a standing central committee and a bureau may be formed to supply graduate students with all the information they require. In the meantime The Hospital will be pleased to supply all information to English graduates, while Professor Kutner of the excellent Committee of the Kaiserin Friedrich Haus in Berlin, will gladly give information regarding courses in Germany and Austria, and the Continent generally, to those who wish to travel farther afield in search of knowledge.

